# Nanostructured Thin Films: Properties, Fabrication and Applications—A Short Review

**DOI:** 10.3390/nano15231760

**Published:** 2025-11-24

**Authors:** Ana-Maria Florea (Raduta), Stefan Caramizoiu, Ana-Maria Iordache, Stefan-Marian Iordache, Bogdan Bita

**Affiliations:** 1 Optospintronics Department, National Institute of Research and Development for Optoelectronics, INOE 2000, 409 Atomistilor, 077125 Magurele, Romania; ana.florea@inoe.ro (A.-M.F.); stefan.caramizoiu@inoe.ro (S.C.); ana.iordache@inoe.ro (A.-M.I.); 2 Department of Electricity, Solid-State Physics and Biophysics, Faculty of Physics, University of Bucharest, 405 Atomistilor, 077125 Magurele, Romania

**Keywords:** nanostructuring, thin-film properties, optoelectronics

## Abstract

Nanostructured thin films are emerging into a diversified class of materials with unique optical, chemical, and physical capabilities as a result of their nanoscale characteristics. This paper provides a complete review of the manufacturing, characterization, and applications of nanostructured thin films in a range of industries such as photonics, electronics, energy storage, and medicine. The relationship between nanostructure morphology and material performance is discussed, as well as the most recent advances in fabrication technologies such as physical vapor deposition, chemical vapor deposition, and solution-based methods.

## 1. Introduction

Usually having a thickness of a few nanometers to several micrometers, nanostructured thin films are a unique class of materials distinguished by their reduced dimensionality and structural characteristics at the nanometer scale [1]. These films have special physical, chemical, optical, and electrical properties that are frequently better than or essentially different from those of their bulk counterparts because they are made up of nanostructured grains, layers, or inclusions. New directions in material science have been made possible by the capacity to precisely manipulate composition, crystallinity, shape, and functional qualities through nanoscale material engineering. The development of cutting-edge technologies, such as optoelectronics [2,3,4,5,6,7,8], sensing devices [9,10,11,12,13], protective coatings [14], energy storage systems [15,16,17], and biomedical applications [18,19], has therefore made nanostructured thin films a key component.

These materials are incredibly adaptable due to their high surface-to-volume ratio, variable porosity, and ability to integrate several functions within a small region. Furthermore, the scalable and repeatable manufacture of nanostructured thin films with customized properties has been made possible by advancements in fabrication processes such atomic layer deposition (ALD) [20,21,22], sol–gel processing [23,24,25], chemical vapor deposition (CVD) [26], and physical vapor deposition (PVD) [27,28].

The need for small, effective, and multipurpose materials in both the industrial and research sectors has fueled an increase in the study of nanostructured thin films in recent years. Such characteristics enable nanostructured materials to deliver enhanced performance in a wide range of applications, from high-efficiency solar cells and rapid-charging batteries to ultra-sensitive sensors and advanced catalysts. Beyond improved performance, nanostructuring allows scientists to tailor material functionalities for specific needs, such as creating water repellent surfaces, antibacterial coatings, and targeted drug delivery systems. As a result, nanostructuring is at the heart of many emerging technologies, including nanomedicine [26,29], quantum computing [30], flexible electronics [31], and next-generation photonic devices [32,33].

The purpose of this review is to present a thorough analysis of nanostructured thin films, emphasizing their properties and production methods. The review is organized so that it begins by outlining the basic properties of nanostructured thin films and then goes into great detail about the several fabrication techniques that are employed to create controlled nanostructures. The effects of nanostructuring on the optical, electrical, chemical, and physical characteristics of thin films are specifically discussed. The analysis concludes by highlighting recent and upcoming uses of these materials in industries like electronics, biomedicine, photonics, and energy, highlighting how nanoscale engineering improves their functionality and performance.

## 2. Properties of Nanostructured Thin Films

Table 1 summarizes the main properties alongside the associated phenomena, physical mechanisms, and theoretical models. This overview provides a clear framework linking nanoscale structure to functional behavior, supporting the discussion of physical, chemical, optical, electrical, magnetic, and defect-related properties in the following sections.

### 2.1. Physical Properties

One of the most distinguishing characteristics of nanoscale materials is their high surface-to-volume ratio, which directly boosts surface energy. Surface atoms have fewer nearby atoms than bulk atoms, resulting in unfulfilled bonds and surplus energy, making the surface thermodynamically unstable. This instability causes surface diffusion, structural reconstructions, and phase shifts at lower temperatures than in bulk materials, increasing reactivity and making nanostructured films ideal catalysts, especially in oxide and metallic thin coatings.

Nanostructured thin films benefit from spontaneous surface restructuring, which occurs particularly under chemical or thermal stress. For example, investigations on ZnO nanostructured thin films have revealed that deposition processes have a considerable influence on film shape and grain size, which affects mechanical, thermal, and catalytic properties [34,35,36,37,38]. In contrast, epitaxial films exhibit smoother surfaces with fewer defects, which reduces surface diffusion but provides superior crystallinity and electronic uniformity, advantageous for high-performance optoelectronics.

**Figure 1:**
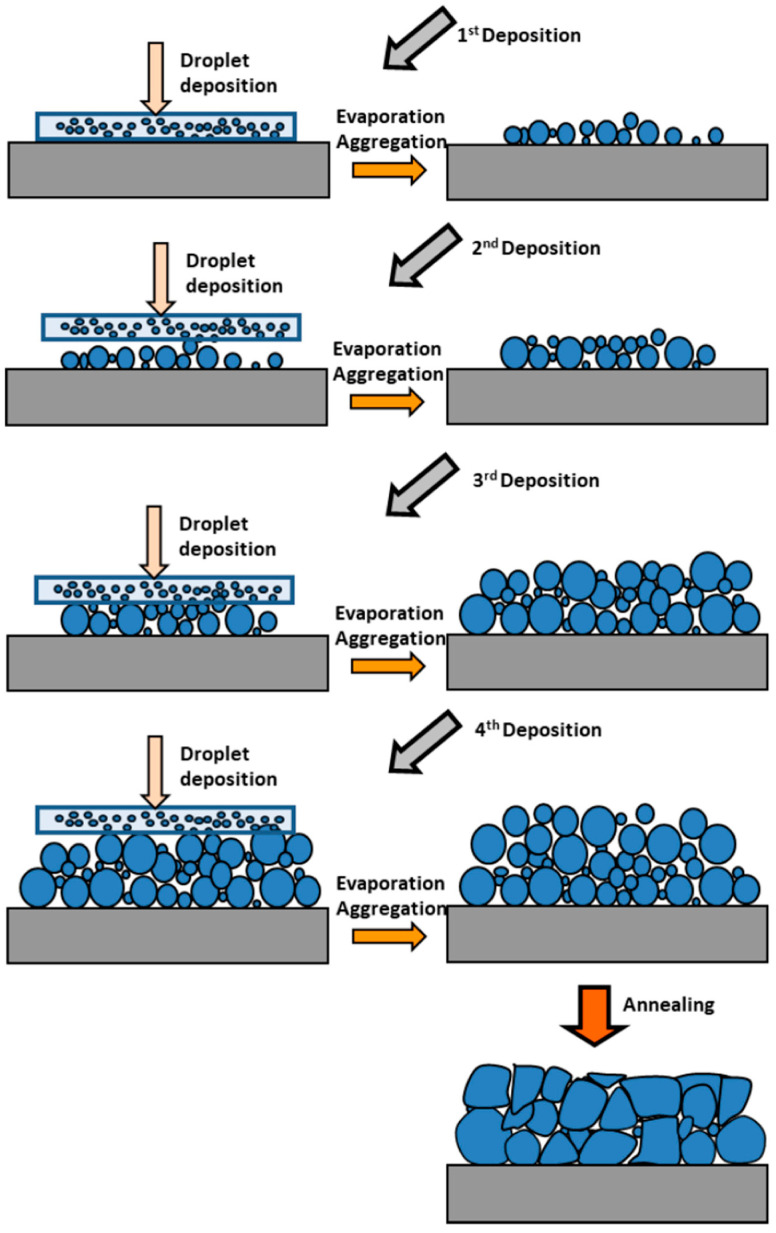
Growth diagram presenting the formation of aggregates during sol–gel spin-coating process [36].

Starting with the first chemical processes that resulted in the production of the ZnO precursor in the solution, the formation of the ZnO film would proceed in the following steps, as schematically illustrated in Figure 1.

At lower temperatures, high surface energy promotes phase transitions and structure reconstructions. FePd thin films, for example, exhibit temperature-induced nanostructured alloy production that is heavily impacted by local surface curvature and high surface-to-volume ratios [39]. Nanostructured films of TiO_2_, CeO_2_, and ZnO have better catalytic activity due to the availability of active surface sites, which may be controlled by shape, porosity, and grain size. Epitaxial films, while less reactive, provide accurate surfaces required for thin-film electronics and optical applications (see Table 2).

### 2.2. Chemical Properties

Nanostructured thin films exhibit enhanced chemical reactivity due to high surface-to-volume ratios, quantum confinement, and reduced dimensionality. The abundance of defect-rich sites accelerates adsorption/desorption kinetics and facilitates surface functionalization for applications in sensors, catalysis, and biomedical interfaces [40,41,42,43,44,45,46,47,48,49]. For example, ZnO and SnO_2_ films are employed in sensitive gas sensors, while nanostructured Pt films improve hydrogen evolution in fuel cells [41]. In comparison, epitaxial thin films offer high chemical stability and uniform surfaces, reducing variability in surface reactions. Protective coatings and microelectronic barriers benefit from epitaxial smoothness, whereas nanostructured films maximize reactive surface area and catalytic efficiency.

### 2.3. Optical Properties

The optical characteristics of nanostructured thin films are strongly impacted by their nanoscale dimensions (see Table 3). In noble metals (Au, Ag), localized surface plasmon resonance (LSPR) causes intense absorption and scattering that can be controlled by particle size, shape, and environment [50,51]. Semiconducting nanostructures exhibit quantum confinement, which alters bandgaps and allows for tunable optical devices like LEDs and solar cells [52,53]. Layer thickness, roughness, and porosity have a further impact on interference, reflectance, and transmittance [54,55]. Advanced characterization techniques, such as UV-Vis-NIR spectroscopy, ellipsometry, PL, FTIR, and Raman, shed light on electronic band structure and plasmonic phenomena [56,57].

Nanostructured films also show improved nonlinear optical properties, such as SHG and SERS, due to localized field amplification around metallic or semiconductor nanostructures [58,59,60,61,62,63,64,65,66,67,68]. For example, Tognazzi et al. [62] demonstrated that TMDC heterostructures exhibit strongly enhanced SHG originating primarily from the interface, where hybrid excitonic resonances and Bound-State-in-the-Continuum (BIC) assisted field localization enable high-Q modes and substantially increase nonlinear susceptibility beyond monolayers or bulk TMDCs. In the domain of ferroelectric oxides, Liu et al. [63] showed that thin-film lithium tantalate (TFLT) can achieve SHG efficiencies comparable to or exceeding bulk materials when film thickness, crystallographic orientation, surface quality, and electrode design are optimized, providing a scalable route for wafer-level fabrication of integrated nonlinear devices. Complementary strategies based on waveguide engineering have also been demonstrated: He et al. [64] reported enhanced SHG in adapted-width, shallow-etched thin-film lithium niobate waveguides by achieving strong mode confinement and phase matching, while Zhao et al. [65] achieved exceptionally high SHG conversion efficiencies (~939%/W) in shallow-etched TFLN waveguides using periodic poling combined with optimized etch depth.

**Table 1:** Theoretical pathway linking material properties to nanoscale phenomena and mechanisms.

Property	Phenomenon	Physical Mechanism	Theoretical Model/Approach	References
Physical Properties	High reactivity, phase transitions at lower T	High surface-to-volume ratio → high surface energy → atomic unsaturation	Surface thermodynamics; Gibbs free energy minimization; surface diffusion models	[34,35,36,37,38,39]
	Grain growth, densification, coarsening	Enhanced surface diffusion during/after deposition	Diffusion models (Arrhenius-type activation), sintering models	[34,35,36,37,38]
	Structural reconstruction	Reduced coordination → lower activation energy for rearrangement	Surface reconstruction theory; thermodynamic stability models	[39]
Chemical Properties	High catalytic activity	Abundant surface active sites (edges, corners, vacancies)	Langmuir–Hinshelwood adsorption kinetics; surface reaction models	[40,41,42,43,44]
	Quantum-size-modified chemical reactivity	Bandgap shifting via quantum confinement	Particle-in-a-box/confinement models	[42,43]
	Ion diffusion, redox behavior	Grain boundary-driven high ion diffusivity	Defect chemistry; diffusion/ion-transport models	[45,46]
	Photochemical activity	Surface defects, charge trapping	Charge-transfer models, defect-state theories	[47,48,49]
Optical Properties	LSPR in metal nanostructures	Collective oscillation of conduction electrons	Mie theory; plasmonics models	[50,51]
	Quantum confinement and bandgap widening	Reduced dimensions < de Broglie wavelength	Effective mass approximation; confinement models	[52,53]
	Interference effects	Thin-film interference (multi-layer reflections)	Fresnel equations; thin-film optical models	[54]
	Scattering, refractive index modification	Surface roughness and porosity	Effective medium theory; scattering theory	[55]
	SHG enhancement	Field localization, exciton–BIC coupling	Nonlinear optics (χ(2) theory); interface-mode modeling	[62,63,64,65]
	SERS enhancement	Electromagnetic field amplification in hot-spots	Plasmonic enhancement models (EM enhancement), charge-transfer theory	[66,67,68,69]
Electrical Properties	Tunable conductivity, transparency	Nanostructure size → percolation pathways	Percolation theory; effective-medium approximations	[62,63,64,65,66,67,68,69,70,71,72]
	Carrier scattering/transport	Grain boundaries as potential barriers	Seto’s grain boundary model; transport scattering models	[72]
	Defect-modulated conductivity	Vacancies, interstitials trap carriers	Polaron models; trap-state theory	[73]
	Thin-film device optimization	Charge transport determined by film thickness/crystallinity	Semiconductor device physics; drift–diffusion models	[74,75,76,77,78,79,80,81,82]
Magnetic Properties	Perpendicular magnetic anisotropy (PMA)	Interface-driven spin–orbit coupling	Magnetic anisotropy models; micromagnetics	[83,84]
	Superparamagnetism	KV ≈ kBT → thermally induced magnet reversal	Néel–Arrhenius model	[85]
	Tunable AFM–FM transition	Doping (Co, Pd, Ir) modifies magnetic phase stability	Phase transition theories; alloy energetics	[86,87,88,89,90]
	Exchange bias	Interfacial uncompensated spins	Meiklejohn–Bean model	[91]
	GMR/TMR	Spin-dependent electron scattering/tunneling	Spin-transport models; Jullière model	[92,93,94,95,96,97]
Defect Engineering in 2D Materials	Defect-induced magnetism	Vacancies/dopants create localized magnetic moments	DFT (first-principles); exchange interaction calculations	[98,99]
	Strain-controlled defect energetics	External strain interacts with local defect fields	Strain–defect coupling models; DFT mechanical simulations	[100,101]
	Gas sensing enhancement	Charge transfer at defect sites	Adsorption energy and charge-transfer models	[100]

**Table 2:** Comparative physical properties of nanostructured and epitaxial thin films.

Property	Nanostructured Thin Films	Epitaxial Thin Films	Implications
Surface energy	High	Low	Reactivity and phase transitions
Grain boundaries	Numerous	Few	Catalytic activity vs. electronic uniformity
Morphology	Tunable via deposition	Smooth, uniform	Morphology control vs. electronic precision
Phase transitions	Lower temperature	Bulk-like	Processing flexibility vs. stability

A similar trend is observed in the development of nanostructured thin films for SERS, where achieving strong electromagnetic “hot spots” and reproducible substrate performance is equally dependent on nanoscale control. Ricci et al. demonstrated that ink-jet printing provides a scalable route to fabricate Au nanostructures with tunable morphology for high-performance SERS and microelectrode integration [66], while Krajczewski et al. reviewed how substrate geometry, material choice, and surface chemistry govern SERS enhancement and reproducibility [60]. Beyond noble metals, Liu et al. advanced semiconductor-based SERS by employing ultrathin WO_3_ films with oxygen-vacancy-induced charge-transfer mechanisms, achieving enhancement factors above 10^6^ and even lower detection limits when hybridized with Au nanoparticles [67]. Visbal et al. further highlighted the environmental relevance of nanostructured Au films by demonstrating their ability to detect water contaminants with high sensitivity and structural uniformity [68]. Additionally, Raj et al. utilized a rapid Dynamic Hydrogen Bubble Template method to produce porous Fe and Pd films, showing that transition-metal nanostructures can also generate strong SERS signals in a cost-effective and scalable manner [69]. Collectively, these studies illustrate that SHG and SERS performance both strongly benefit from nanoscale structural design, with nanostructured thin films offering versatile, high-efficiency platforms for nonlinear photonics and sensing applications, as can be seen from Table 3.

**Table 3:** Nanoscale optical responses and engineering in nanostructured versus epitaxial thin films.

Optical Effect	Nanostructured Films	Epitaxial Films	Applications
LSPR	Strong, tunable	Weak	Sensing applications
SHG	Enhanced via hot spots	Moderate	Nonlinear photonics
SERS	High enhancement	Limited	Substrate uniformity vs. signal
Quantum confinement	Tunable bandgap	Bulk-like	LEDs, photovoltaics

### 2.4. Electrical Properties

The charge transport in nanostructured thin films is adjustable related to grain size, interface density, and defect concentration [70,71,72,73,74,75,76,77,78,79,80,81,82]. Optimizing percolation routes allows for excellent conductivity while maintaining transparency in flexible electronics [80]. Depending on the design, grain boundaries and defect states can scatter carriers or allow for charge trapping.

Epitaxial films, on the other hand, provide high carrier mobility and uniform conductivity as a result of reduced defects, making them ideal for high-performance transistors and photodetectors [83,84,85,86,87,88,89,90,91]. Recent techniques, such as scanning Kelvin probe microscopy (SKPM) and conductive AFM, enable the mapping of electrical characteristics at the nanoscale to compare epitaxial and nanostructured films.

### 2.5. Magnetic Properties

Nanostructured thin films display emergent magnetic phenomena caused by finite-size effects, increased surface-to-volume ratios, quantum confinement, and interfacial exchange interactions. Key magnetic parameters, such as coercivity (Hc), magnetic anisotropy (K), saturation magnetization (Ms), Curie temperature (Tc), and domain-wall dynamics, can be precisely controlled by deposition conditions, film thickness, grain size, crystallographic texture, strain states, and interfacial chemistry [92,93]. The transition from in-plane to perpendicular magnetic anisotropy (PMA) in ultrathin films is a noticeable effect caused by spin–orbit coupling, surface/interface anisotropy, and magnetoelastic interactions. Systems like Co/Pt, Co/Pd, and Fe/MgO show how nanoscale engineering allows for thermally stable, high-density magnetic storage in MRAM and racetrack memory [92,93]. At critical thicknesses or particle sizes, superparamagnetism occurs when the effective anisotropy energy (KV) approaches thermal energy (kBT), causing spontaneous magnetization reversal and hysteresis collapse [94]. This sets a fundamental limit for magnetic storage at the nanoscale. Dopant engineering in FeRh-based systems can affect the antiferromagnetic-to-ferromagnetic (AFM-FM) transition temperature (Tt). For example, Co doping in FeRh thin films reduces Tt and improves ferromagnetic stability at low temperatures [96,97], whereas Pd/Ir compositional gradients enable smooth, controllable AFM-FM phase transitions across a wide temperature range [98,99]. These studies demonstrate how chemical composition and interface control can influence magnetic behavior.

Spin-dependent electron scattering can also be observed in nanostructured multilayers and granular films, such as giant magnetoresistance (GMR) and tunneling magnetoresistance (TMR) [101,102,103]. Recent discoveries include atomic layer deposition (ALD) of sub-nanometer Al_2_O_3_ tunnel barriers and integration of two-dimensional van der Waals materials into MTJs, producing increased TMR values (~77–90% at ambient temperature and 100 K) and numerous nonvolatile resistance states [104,105,106]. Epitaxial magnetic films outperform nanostructured films in terms of crystallographic order and reproducibility, allowing for fine control of anisotropy, coercivity, and magnetic phase transition. Nanostructured films, on the other hand, provide increased tunability and better interfacial effects, making them ideal for sensors, spintronic devices, and magnetic switching applications.

### 2.6. Defect Engineering in Two-Dimensional (2D) Materials

Defect engineering allows for tuning of electrical, optical, and magnetic properties in 2D materials such as MoS_2_, WS_2_, and h-BN, as summarized in Table 4 [107,108,109,110]. Controlled vacancies, dopants, and grain boundaries produce magnetism, alter bandgaps, and enhance gas sensing. Strain and defect interactions fine-tune material behavior, creating a versatile platform for spintronics, sensors, and flexible electronics.

Nanostructured films have a greater impact on defect engineering due to larger surface areas and customizable morphologies, whereas epitaxial 2D films ensure crystallographic homogeneity, providing consistent baseline attributes for device manufacturing.

## 3. Fabrication and Characterization Methods of Nanostructured Thin Films

Nanostructured thin-film production is critical to the advancement of applications in electronics, optics, energy storage, and biomedical device technology. Nanostructured films have greater tunability in shape, surface area, porosity, and defect density than epitaxial films, which has a direct impact on their physical, chemical, optical, and electrical properties. While epitaxial films have greater crystallographic order and well-defined interfaces, they are often grown on lattice-matched substrates under high-temperature processing and controlled vacuum conditions, which can limit scalability and cost-efficiency. Nanostructured films, on the other hand, can typically be deposited utilizing simpler, lower-temperature, and less expensive processes while still achieving improved functional performance. Atomic Layer Deposition (ALD) enables atomic-scale thickness control and superior conformality, which are essential for both nanostructured and epitaxial films. ALD may cover high-aspect-ratio 3D templates, such as ZnO nanostructures, in sub-5 nm mesoporous silica, allowing for exquisite morphological control [111]. Epitaxial ALD films benefit from higher crystallinity and interface coherence, which improves electrical and optical transport, but they are less adaptive to non-planar or porous templates [111,112].

Solution-based approaches, such as the sol–gel process, are very useful for nanostructured films because they allow for the incorporation of nanomaterials such as quantum dots while also tailoring optical/electronic properties. For example, PbS-doped inorganic films generated using sol–gel exhibit improved photoluminescence for optoelectronic applications [25]. Epitaxial films, in contrast, necessitate lattice matching and regulated crystallization conditions, making low-cost solution processing difficult. Physical vapor deposition (PVD) technologies, such as magnetron sputtering, allow for the deposition of nanostructured films with controllable stoichiometry, controlled porosity, and changeable surface roughness, thereby improving catalytic, sensing, and optical performance [113,114,115]. PLD can create nanostructured and epitaxial films.

Nanostructured ZnO and TiO_2_ films have higher surface area and photocatalytic activity [116], while epitaxial BiFeO_3_ and SrTiO_3_ films have high crystallinity and reproducibility, making them ideal for ferroelectric or multiferroic applications [117]. Nanostructured films frequently provide greater flexibility in phase tuning and defect engineering during deposition, which is beneficial for device optimization. Emerging approaches such as Electrostatic Spray-Assisted Vapor Deposition (ESAVD) emphasize the scalability and adaptability of nanostructured film deposition, in contrast to the more stringent epitaxial growth requirements [118]. Nanostructured films require advanced characterization to capture their distinct structural, chemical, and functional characteristics. Surface chemistry, defect evolution, and interface dynamics can be monitored in real time using in situ and operando techniques like as X-ray photoelectron spectroscopy (XPS), environmental TEM, and scanning electrochemical microscopy (SECM). These approaches are especially useful for nanostructured films, where high surface-to-volume ratios, defect-rich regions, and heterogeneous active sites result in dynamic behavior that is not normally seen in epitaxial films [119,120,121,122].

Morphological and crystallographic evaluation with XRD, AFM, and SEM reveals clear distinctions: epitaxial films typically have sharp diffraction peaks, low roughness, and highly oriented grains, whereas nanostructured films have broadened peaks, tunable roughness, and intrinsic porosity, which improve adsorption, catalytic reactivity, and photon-matter interactions [122]. Electrical and electrochemical measurements (four-point probe, Hall effect, and cyclic voltammetry) show that epitaxial films have higher carrier mobility and less defect scattering, whereas nanostructured films use large interfacial areas and engineered defects to improve electrochemical performance and sensing characteristics. Magnetic techniques such as VSM, SQUID, and MOKE demonstrate that nanostructuring allows for variable magnetic anisotropy, coercivity, and defect-mediated phase behavior, whereas epitaxial films maintain uniform and stable magnetic responses with little grain-boundary effects [122].

## 4. Advancements and Applications

### 4.1. Energy Conversion and Storage

**Figure 2:**
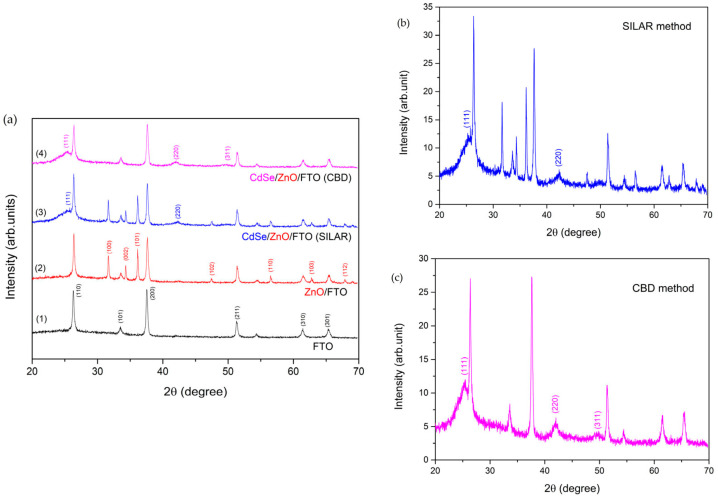
X-ray diffraction patterns of bare FTO ((**a**), 1 curve), ZnO/FTO ((**a**), 2 curve), CdSe/ZnO/FTO (SILAR) ((**a**), 3 curve and (**b**)), and CdSe/ZnO/FTO(CBD) ((**a**), 4 curve and (**c**)) [124].

Recent advancements in nanostructured thin films have significantly propelled the fields of energy conversion and storage, offering innovative solutions for next-generation devices. These materials, characterized by their ultra-small building blocks and high interface-to-volume ratios, enable enhanced energy conversion efficiency and power density. By creating polar nanoregions using low-energy ion implantation, for example, scientists have tripled the energy storage density of PbZrO_3_ thin films, increasing it from 20.5 J/cm^3^ to 62.3 J/cm^3^ [123]. Cadmium selenide/zinc oxide (CdSe/ZnO) thin films were fabricated on Fluorine-doped Tin Oxide (FTO) substrates using low-temperature chemical bath deposition (CBD) and (successive ionic layer adsorption and reaction) SILAR methods, both low-cost and scalable. In Figure 2, the X-ray diffraction (XRD) confirmed hexagonal ZnO and cubic CdSe phases, with crystallite sizes of 44 nm (ZnO) and 10 nm (CdSe/ZnO) [124].

Scanning Electron Microscopy (SEM) and Atomic Force Microscopy (AFM) showed that CBD films had denser, more uniform structures, while UV–Vis spectroscopy revealed band gaps of 3.2 eV (ZnO) and 1.85–1.97 eV for CdSe/ZnO. Elemental analysis verified stoichiometric CdSe deposition. Notably, SILAR derived films exhibited superior photocatalytic and optoelectronic performance [124].

**Figure 3:**
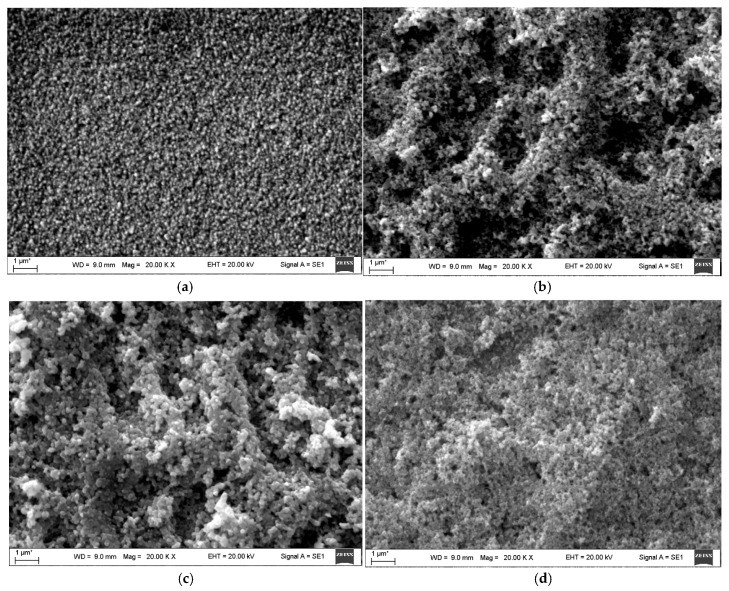
SEM images of pristine FTO (**a**), ZnO/FTO (**b**), and thin films of CdSe obtained by SILAR (**c**) and CBD (**d**) [124].

Figure 3 presents SEM images of pristine FTO and ZnO-coated FTO substrates. The bare FTO surface (Figure 3a) displays uniformly distributed fine crystallites. Following ZnO nanoparticle deposition, the surface morphology changes markedly, forming a novel porous and reticulated structure of uniformly sized crystallites (Figure 3b), indicating complete and uniform ZnO coverage. Additionally, the SEM images reveal the morphology of CdSe/ZnO thin films produced via different CdSe deposition methods. Films fabricated by CBD (Figure 3d) exhibit a denser, more compact structure with superior crystallinity compared to those prepared by the SILAR method (Figure 3c).

A notable example is the use of nanostructured TiO_2_ thin films, which, by combining with plasmonic aluminum and gold metal nanostructures, have demonstrated increased optical absorption in the visible and near-infrared spectrum, leading to established photocatalytic activity, useful in wastewater treatment [125].

Recent advances in perovskite solar cells have focused on stability, interface engineering, and crystallization control to push efficiencies closer to the theoretical limit. Li et al. [126] demonstrated that incorporating dipeptide molecules into MAPbI_3_ perovskites significantly improved both efficiency and operational stability by passivating defects and enhancing film quality. In parallel, Hou et al. [127] reported a simulation study of HTL-free CsPbI_3_/MAPbI_3_ heterojunctions, achieving a remarkable predicted efficiency of 30.33%, suggesting that interface optimization alone can reduce recombination losses and simplify device architecture. Complementarily, Chen et al. [128] achieved controlled crystallization of the metastable γ-CsPbI_3_ phase via methylammonium iodide-assisted co-evaporation, which yielded enhanced film uniformity and improved photovoltaic performance.

MXene-based materials have emerged as highly promising candidates for next-generation energy storage systems, particularly in supercapacitors, due to their exceptional electrical conductivity, tunable surface chemistry, and layered structure. Ahmad and Oh [129] highlighted recent developments in MXene composites, emphasizing their dual role in supercapacitors and electrochemical sensing, where surface functionalization and hybrid architectures have markedly improved capacitance and stability. Earlier, Miao et al. [130] summarized the potential of novel MXene materials in enhancing energy storage density, pointing to their superior charge transport and structural advantages over conventional carbon-based electrodes. More recently, Hu et al. [131] provided a theoretical framework, detailing the principles of MXene supercapacitors and demonstrating their integration into power electronic systems, underlining their capacity to deliver rapid charge–discharge cycles and high power density.

**Figure 4:**
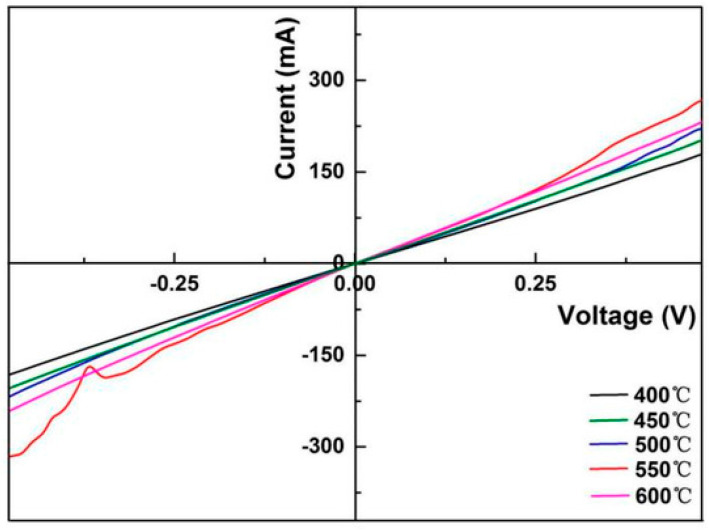
I–V curves of CZTS/Ti structures heat-treated at different sulfurization temperatures [132].

The family of kesterite semiconductors, particularly Cu_2_ZnSnS_4_ (CZTS) and Cu_2_ZnSnSe_4_ (CZTSe), has emerged as a sustainable alternative to traditional CIGS absorbers for thin-film solar cells. Their appeal lies in the abundance and non-toxicity of constituent elements, combined with suitable band gaps and strong absorption coefficients. Huang et al. investigated the role of titanium (Ti) substrates in enhancing the performance of flexible CZTS thin films [132]. They demonstrated that sulfurization temperature critically controls Ti diffusion into the absorber layer. At 550 °C, the films exhibited higher base-grain density, suppressed Ti diffusion, and improved ohmic contact, resulting in optimized current–voltage behavior, as shown in Figure 4.

Figure 4 presents the I–V curves of CZTS/Ti structures prepared at different sulfurization temperatures. All curves are linear, confirming ohmic contact between CZTS films and Ti substrate. The slopes increase from 0.37 S (400 °C) to 0.55 S (550 °C), then decrease to 0.50 S (600 °C). Thus, 550 °C yields the best ohmic contact with lowest resistance, favoring carrier transport. This optimal behavior is likely due to suitable Ti diffusion into the CZTS films.

**Figure 5:**
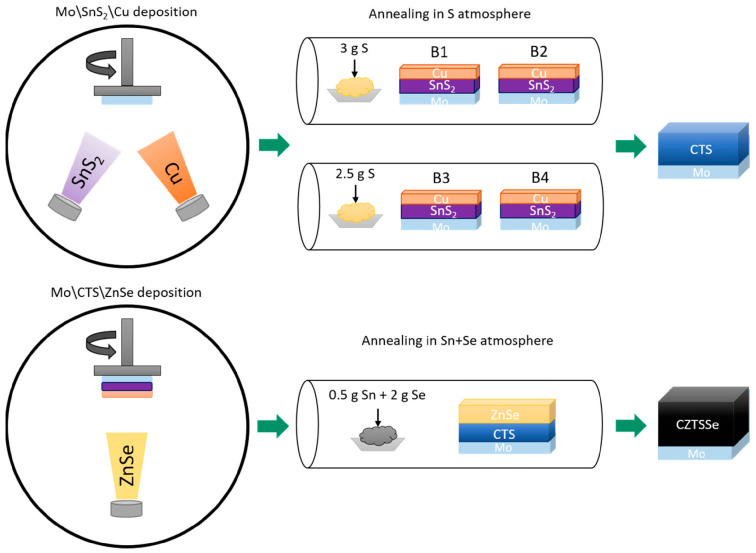
Schematic diagram of the deposition and annealing processes [133].

Further progress has been achieved in bandgap engineering by tuning the sulfur/selenium ratio in quaternary alloys. Zaki et al. [133] demonstrated a two-step magnetron sputtering and annealing process to synthesize CZTSSe films with controlled S/(S + Se) ratios ranging from 0.83 to 0.44 (Figure 5).

Decreasing sulfur content led to larger grain sizes, improved densification, and caused a compositional transition from CZTS-like to CZTSe-like phases, while maintaining single phase purity. Such tunability allows tailoring the absorber bandgap between 1.0 eV and 1.5 eV, balancing carrier mobility and absorption for improved photovoltaic conversion.

A novel two step synthesis technique for MoS_2_ thin films comprises sulfurization in a confined graphite box after sequential deposition of Mo and MoS_2_ precursor layers using magnetron sputtering [134]. This approach reduces toxic gases such as SO_2_ and prevents the use of poisonous H_2_S. It also significantly reduces the quantity of sulfur required by up to an order of magnitude. The process enables safer and more sustainable fabrication of nanostructured MoS_2_ thin films on Si/SiO_2_ substrates, resulting in environmentally friendly energy storage devices. Recent studies on electrocatalysts and photocatalysts for hydrogen evolution reactions based on two-dimensional materials, primarily MoS_2_, WS_2_, and related compounds, are compiled in [135]. A summary and prospecting of the difficulties and future prospects of development for electrocatalysts and photocatalysts of two-dimensional hydrogen evolution reactions are also provided.

For the large area development of uniform 2D transition metal dichalcogenide (TMD) films, a scalable physical deposition technique has been developed [136]. By successively stacking few-layer WS_2_ and MoS_2_, this technique makes it possible to fabricate van der Waals heterostructures. This results in a type-II heterojunction that performs better photocatalytically than MoS_2_ alone. The device, which combines a gold top contact with a graphene bottom electrode, shows photovoltaic and photocurrent under light, demonstrating the potential of this large-scale growth strategy for self-powered photoconversion applications.

Liu Y. et.al. reported a simple and cost-effective cathodic deposition technique for fabricating nanostructured V_2_O_5_ thin films using a solution of V_2_O_5_ and H_2_O_2_ [137]. The resulting films exhibit a distinctive nanostructure that enhances phase transitions during lithium-ion insertion, leading to high energy and power densities along with excellent cycling stability when used as thin-film cathodes in Li-ion batteries. It has been reported that RF-sputtered V_2_O_5_ thin films, followed by furnace annealing, exhibit significantly enhanced electrochromic performance [138]. After annealing at 400 °C, the films demonstrated improved charge capacity (97.9 mC/cm^2^), optical transmittance difference (31%), and coloration efficiency (6.3 cm^2^/C). These improvements were attributed to the formation of a polycrystalline orthorhombic structure, which promotes lithium-ion intercalation and increases charge storage capacity, making the films suitable for use as ionic storage layers in electrochromic devices.

Recent work has demonstrated that V_2_O_5_/ZnO thin films, deposited on flexible substrates via magnetron sputtering with varying radio power, can serve as effective electrode materials for transparent energy storage devices [139]. The study showed that adjusting the sputtering power influenced film thickness and optical transmittance, with optimized films at 80 W achieving an areal capacitance of 83.59 mF/cm^2^, 95.18% capacitance retention after 5000 cycles, and 70% transparency. Furthermore, a fabricated transparent symmetric supercapacitor based on these films delivered an areal energy density of 0.46 μWh/cm^2^ at a power density of 62 μW/cm^2^, retaining 75.41% of its capacitance after 6000 cycles.

### 4.2. Smart Coatings and Self-Cleaning Surfaces

**Figure 6:**
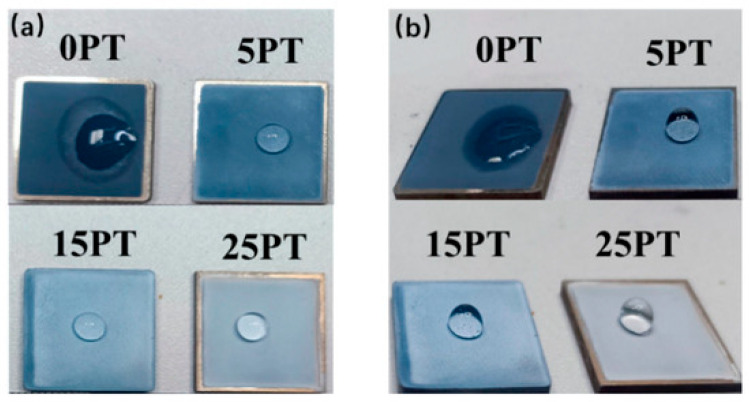
Top view (**a**), and side view (**b**), of the hydrophobic effect of the sample [141].

ZnO nanostructured thin films with varied morphologies were grown on glass using a simple two step wet chemical method [140]. ZnO seed layers were deposited by SILAR at 80 °C, followed by hierarchical ZnO nanostructures via chemical bath deposition. Deposition time and pH-controlled film shape, crystallinity, optical band gap (2.45–3.62 eV), and wettability, shifting from hydrophilic to hydrophobic (contact angle up to ~135°). These stable hydrophobic films show promise for self-cleaning and gas sensing applications. A recent study reported the development of a novel hydrophobic, photocatalytic, self-cleaning composite coating by combining photoactive TiO_2_ with hydrophobic perfluoroalkoxy (PFA) [141]. Using suspension plasma spraying, TiO_2_ PFA coatings were successfully deposited over large surface areas, offering an effective self-cleaning solution without the complexity associated with chemical processing methods (see Figure 6). Morphological analysis revealed that the coatings exhibit numerous circular and ellipsoidal nanoparticles embedded within a flocculent, porous micro/nano-structured matrix, attributed to the presence of PFA. Phase characterization via XRD confirmed that the coatings predominantly consist of anatase phase TiO_2_, alongside rutile and (C_2_F_4_)ₙ from the PFA component.

Figure 6 shows the top-view and side-view of water droplets on the coating surface, visually confirming the strong hydrophobic behavior created by adding PFA. This is a key result of the study, since the combination of hydrophobicity and photocatalytic activity is what makes these coatings effective for practical self-cleaning applications.

For example, vanadium dioxide (VO_2_) demonstrated significant potential for self-cleaning applications through its thermochromic and photochromic properties [142]. Recent studies reported the low-temperature (320 °C) deposition of thermochromic V_1−x−γ_W_x_Sr_γ_O_2_ films with thicknesses of 71–73 nm onto Y-stabilized ZrO_2_ layers measuring 170–175 nm, which were themselves deposited on conventional 1 mm thick soda-lime glass substrates [143]. The deposition process employed reactive high-power impulse magnetron sputtering coupled with pulsed O_2_ flow feedback control, enabling the preparation of crystalline W and Sr-co-doped VO_2_ films with precise stoichiometry, without the need for substrate bias or post-deposition annealing. Tungsten doping effectively reduced the transition temperature below 25 °C, whereas strontium doping significantly increased the integral luminous transmittance (T_lum_) by widening the optical band gap in the visible range, consistent with a reduction in the films’ absorption coefficient. The influence of Sr content within the metal sublattice on the electronic and crystal structure of V_1−x−γ_W_x_Sr_γ_O_2_ films, as well as on their temperature-dependent optical and electrical properties, was thoroughly investigated. An optimized composition, V_0.855_W_0.018_Sr_0.127_O_2_, demonstrated a high T_lum_ of 56.8% and solar energy transmittance modulation (ΔT_sol_) of 8.3%, representing improvements of 1.5 and 1.28 times, respectively, compared to V_0.984_W_0.016_O_2_ films. These findings marked a significant advancement toward the low-temperature synthesis of large-area thermochromic VO_2_-based coatings suitable for smart window applications. Moreover, it was shown that T_lum_ and ΔT_sol_ could be further enhanced by over 6% and 3%, respectively, through the application of a 280 nm thick SiO_2_ antireflection top layer.

### 4.3. Sensing and Biomedical Applications

Stramarkou et.al. [23] reported the fabrication of sensors which can detect modifications in CO_2_ concentrations at room temperature, thus indicating the quality or microbial spoilage of food products when incorporated into food packaging. ZnO nanostructures are known for their ability to detect organic gases; however, their effectiveness is limited to high temperatures (greater than 200 °C). To overcome this limitation, sodium (Na) doping was investigated as a way to enhance the sensing properties of ZnO films and lower the working temperature. In this study, undoped and Na-doped ZnO thin films were developed via the sol–gel method with different Na percentages (2.5, 5 and 7.5%) and were deposited via spin coating. The crystal structure, the morphology, and the surface topography of the developed films were characterized by X-ray Diffraction (XRD), Scanning Electron Microscopy (SEM), and Atomic Force Microscopy (AFM), respectively. Furthermore, the response to CO_2_ was measured by varying its concentration up to 500 ppm at room temperature. All the developed films presented the characteristic diffraction peaks of the ZnO wurtzite hexagonal crystal structure. SEM revealed that the films consisted of densely packed grains, with an average particle size of 58 nm. Na doping increased the film thickness but reduced the surface roughness. Finally, the developed sensors demonstrated very good CO_2_ sensing properties, with the 2.5% Na-doped sensor having an enhanced sensing performance concerning sensitivity, response, and recovery times. This leads to the conclusion that Na-doped ZnO sensors could be used for the detection of microbial spoilage in food products at room temperature, making them suitable for smart food packaging applications.

**Figure 7:**
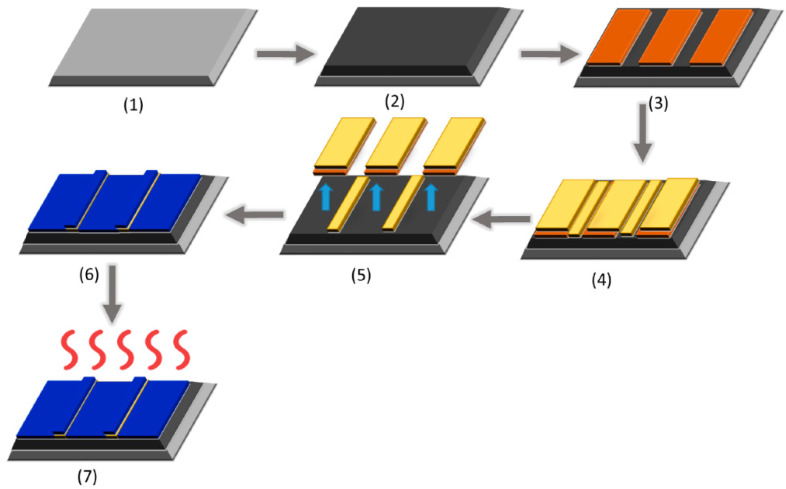
Fabrication workflow of the CO_2_ sensor: (1) Si wafer cleaning (piranha solution), (2) thermal oxidation, (3) negative lithography, (4) Ti/Au electrode deposition, (5) lift-off, (6) ZnO thin-film coating, and (7) final annealing. Color code: light gray = Si wafer, dark gray = oxidized Si, orange = Au, yellow = Ti/Au, blue = ZnO [23].

Figure 7 shows a schematic sequence of the fabrication process steps. The process began by cleaning a four-inch n-type Si (100) wafer with a piranha solution to remove organic contaminants, followed by rinsing and drying (Figure 7, Step 1) [23]. A 100 nm SiO_2_ layer was grown via dry thermal oxidation and cleaned again (Step 2). Negative lithography was performed by spin-coating a 1.3 μm AZ-5214E photoresist, baking, UV exposure using a mask aligner, post-bake, flood exposure for image reversal, and developing (Step 3). A 10 nm Ti adhesion layer and 50 nm Au layer were deposited via DC magnetron sputtering (Step 4), followed by a lift-off process in acetone to define interdigitated electrodes (Step 5). The wafer was then cleaved into samples. ZnO thin films were deposited by spin-coating (seven cycles at 2000 rpm, 30 s), with thermal treatments after each layer and final annealing at 500 °C (Steps 6–7).

SnO_2_–ZnO heterostructures fabricated by RF magnetron sputtering exhibit significantly improved NO_2_ detection at low concentration (~0.1 ppm), attributed to heterojunction-induced surface electric fields up to 2 × 10^7^ V/cm, enhancing surface sensitivity [143,144]. Thin films of aluminum-doped zinc oxide (AZO) were deposited via RF magnetron sputtering for application in CO gas sensing. By adjusting the oxygen flux and sputtering power, the porousness of the film surface was optimized to enhance sensing performance. An oxygen flux of 10 sccm and a sputtering power of 175 W yielded the highest response value of 1.138, representing a 13% improvement compared to untreated films. These results confirm that introducing controlled oxygen during deposition and increasing sputtering power effectively promote film porosity, thereby improving gas sensitivity. Notably, this sensor demonstrated efficient gas-sensing behavior at an operating temperature as low as 100 °C, outperforming many existing oxide-based sensors that require higher temperatures. Further microstructural analysis of the optimized AZO films is ongoing to clarify the correlation between surface morphology and gas-sensing behavior, with promising implications for low-temperature, high-performance gas sensor applications [145]. The current landscape of gas sensors developed via magnetron sputtering for volatile organic compound (VOC) detection was examined by Moura, P.C. and Sério, S [10]. Analysis of key studies revealed that sensors fabricated by this technique primarily target ten major VOCs, with thirteen additional compounds identified as emerging priorities. The influence of critical sputtering parameters—such as power, pressure, substrate temperature, gas flow, and deposition time on thin-film properties and sensor performance was systematically outlined. Among the metal oxides, ZnO and TiO_2_ were the most widely used, with WO_3_, CuO, and SnO_2_ also extensively investigated. The incorporation of noble metal dopants was found to enhance sensitivity and selectivity. Future research directions include the use of nanostructured and hybrid materials, dynamic sputtering control, multilayer structures, and advanced surface functionalization to improve sensitivity, selectivity, durability, and real-world applicability.

Metal oxide thin films, including copper oxide (CuO), titanium dioxide (TiO_2_), and tin dioxide (SnO_2_), were deposited via magnetron sputtering and employed as gas-sensitive layers in microwave-based gas sensors operating at 2.4 GHz [146]. The sensors were evaluated at room temperature (23 °C) and 50% relative humidity under exposure to 0–200 ppm concentrations of selected VOCs, namely acetone, ethanol, and methanol, relevant to industrial and biomedical applications. The results demonstrated that CuO-based sensors exhibited the highest response to acetone, SnO_2_ based sensors showed superior sensitivity to ethanol, and both SnO_2_ and TiO_2_ thin films displayed notable sensitivity to methanol.

**Figure 8:**
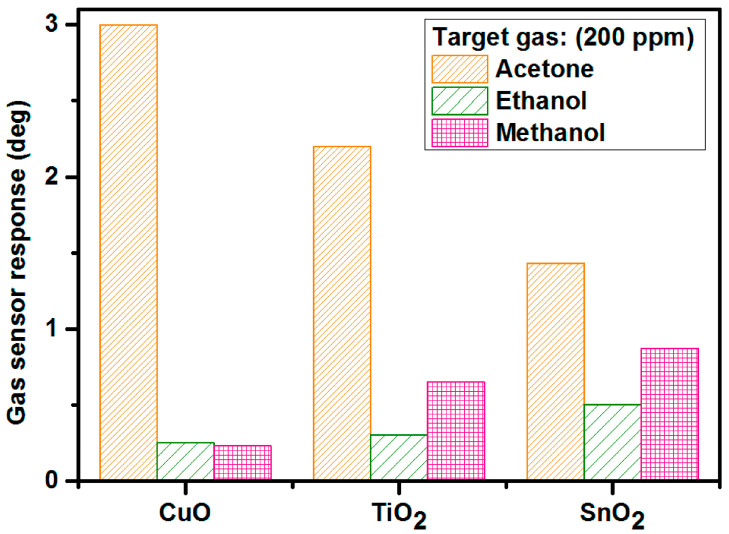
Gas-sensor response to acetone, ethanol, and methanol at room temperature, 50% relative humidity, and 200 ppm for CuO-based, TiO_2_-based, and SnO_2_-based microwave gas sensors [146].

Figure 8 shows the gas-sensor response values of CuO-based (250 nm), TiO_2_-based (200 nm), and SnO_2_-based (250 nm) microwave gas sensors exposed to several volatile organic compounds: acetone, ethanol, and methanol. The target gas concentration, measurement temperature, and relative humidity levels were as follows: 200 ppm, 23 °C, 50%. CuO-based microwave gas sensors had the strongest reaction to acetone (~3), followed by ethanol (~0.87) and methanol (~0.5) for SnO_2_-based sensors. Although the obtained phase discrepancies were not great, they were at quantifiable levels, with a noise standard deviation of around 0.04°. Copper oxide as a gas-sensitive layer has a high sensitivity to acetone but almost negligible sensitivity to ethanol and methanol (below 0.3 deg of phase shifts), making this metal oxide an appealing gas-sensing material in microwave applications. Titanium dioxide and tin dioxide were employed to detect methanol at the same response level [146].

Tripathy et al. [147] developed a room-temperature CO_2_ sensor using layered composites of polyethylenimine (PEI), nitrogen-doped reduced graphene oxide (NrGO), and vertically aligned ZnO nanorods (ZNRs). The 3D PEI/NrGO network supported by ZNRs enhances CO_2_ adsorption while preventing agglomeration, resulting in a strong, linear response, excellent stability, and reproducible performance. The sensor operates via CO_2_ adsorption and subsequent formation of carbamate and carbonic acid, demonstrating its potential for practical, non-invasive CO_2_ monitoring applications. In 2024, Haldar et al. [148] used p–p-type heterostructures made from metal–organic frameworks (MOFs) to provide a high-performance CO_2_ sensor that functions at ambient temperature. With a low detection limit of 2 ppm, the sensor demonstrated a notable response to CO_2_ concentrations ranging from 39.6 to 500 ppm at 25 °C by integrating copper oxide (CuO) nanoparticles into reduced graphene oxide (rGO) sheets. Over the course of 30 days, the sensor maintained 98% of its initial performance, demonstrating exceptional long-term stability. It also continued to function when the relative humidity exceeded 40%. Understanding of the sensor’s functioning was improved by first-principles simulations that shed light on the mechanics behind interactions between the sensor and CO_2_ molecules.

The frontiers of nanostructured thin films for FET and electrochromic biosensors have rapidly advanced through innovative 2D material engineering and device integration. A comprehensive review reported by Moore & More underscores the pivotal role of graphene, Transition Metal Dichalcogenides (TMDs) (e.g., MoS_2_, WS_2_), and MXenes in boosting electrochromic device performance, enabling multifunctional biochemical sensing with enhanced ion diffusion, rapid optical switching, and robust mechanical flexibility [149].

### 4.4. Future Outlook and Challenges

The future of nanostructured thin films depends on managing difficult trade-offs between sustainability, scalability and commercialization, digital/AI-assisted optimization, and the development of new materials. Functional limits are being pushed by recent developments in materials like MXenes, perovskites, and 2D heterostructures. For instance, MXenes have attracted a lot of interest in energy storage, sensing, and flexible electronics because of their high conductivity, mechanical resilience, and tunable surface terminations [150,151]. However, issues including oxidation, restacking, large-scale production, and precise interface control continue to be significant barriers to deployment (molten salt etching techniques, for example, have been suggested to enhance scalability and termination control in MXenes). Although perovskite thin films continue to exhibit remarkable optoelectronic performance (see Table 5), their potential for long-lasting devices is still constrained by concerns about environmental resilience and long-term stability (moisture, ion migration, and light-induced deterioration).

Emerging design workflows are increasingly leveraging machine learning and data-driven analytics to optimise thin-film fabrication, structure, and performance. For example, ML-assisted analysis of RHEED video data enabled classification of growth modes in 2D TMDC thin films, leading to improved control over defect formation and crystallinity [156]. In contrast to conventional simulation-driven techniques, neural-network-based inverse design has been used to achieve desired reflectance/transmittance profiles in the optical domain on multilayer nano-thin films [157]. Furthermore, a sizable experimental dataset that included failures allowed an ML model to predict which monomer pairings would create free-standing films in polymer-based composite thin films, demonstrating the importance of negative data in directing thin-film design [158]. For example, the Daisy Visual Intelligence Framework utilizes AI models to propose new synthesis conditions based on historical microscopic images, facilitating the design of thin films with desirable microstructures [159].

Wang et al. [160] present an autonomous AI-driven platform (“Polybot”) for solution processing of electronic polymer thin films. The system integrates robotic material handling, in situ characterization, and machine learning optimization in a closed-loop workflow. By exploring a vast multidimensional parameter space, Polybot autonomously identifies optimal processing conditions for polymer films such as PEDOT:PSS, achieving conductivities above 4500 S cm^−1^ and excellent uniformity.

Green synthesis and sustainability are also becoming essential since they are influencing the development of thin-film fabrication in the future. Solvent engineering, biomass-derived reducing agents, low-temperature photonic curing, and renewable precursors are the main areas of recent work. A hybrid transparent conducting electrode was created on PET using photonic curing and blade coating, which resulted in ~11 Ω/sq sheet resistance and ~81% transmittance while reducing thermal budget and carbon emissions in comparison to traditional sintering methods [161].

Simultaneously, the use of plant extracts (such as Camellia sinensis and Neem) in green-chemistry assisted synthesis has made it possible to embed metal nanoparticles into polymer films with a longer lifespan (>18,500 h) under safe processing circumstances [162,163] “Greener” thin-film composite membranes with nanofillers have been investigated for membrane systems in order to reduce energy costs and improve stability under actual feed streams.

Overall, tailoring nanostructured thin films involves a range of approaches, such as controlling porosity, grain size, defects, interfaces, and thickness to optimize optical, electronic, magnetic, catalytic, and surface properties. These strategies often influence multiple functional responses simultaneously, highlighting the need for careful trade-offs between performance, stability, and scalability. To provide a concise overview of these methods and their observed effects, Table 6 summarizes key approaches for tailoring thin-film properties and the resulting outcomes.

## 5. Conclusions

In this study, we have emphasized the rapidly developing subject of nanostructured thin films, emphasizing its tremendous potential in a wide range of applications. By comparing these materials to epitaxial thin films, we have demonstrated that nanoscale structuring brings novel optical, electrical, mechanical, and surface-driven features that differ dramatically from those achieved in highly ordered epitaxial systems. While epitaxial thin films have superior crystalline quality, well-defined interfaces, and excellent charge-transport properties, nanostructured thin films have enhanced tunability, increased surface area, and multifunctionality—all of which are particularly useful for applications in energy conversion and storage, smart coatings, self-cleaning surfaces, and biomedical sensing.

The continual development of manufacturing methods, ranging from traditional physical and chemical deposition approaches to emergent techniques like atomic layer deposition and solution-based processes, is broadening the design space for both nanostructured and epitaxial designs. However, the adaptability and scalability of nanostructured thin films make them ideal for incorporation into next-generation devices tackling difficulties in sustainability, healthcare, and advanced manufacturing.

From our perspective, the most intriguing potential is found in combining the complimentary characteristics of nanostructured and epitaxial thin films. Hybrid or hierarchical systems that combine high crystalline order with nanoscale functional characteristics have the potential to create devices that can harvest energy, sense the environment, and self-adapt to operating circumstances all at once. Furthermore, advances in cost-effective, environmentally friendly, and scalable production procedures will be critical for moving these materials—particularly the more flexible nanostructured platforms—from laboratory demonstrations to widespread industrial use.

## Figures and Tables

**Table 4:** Defect engineering strategies and functional effects in two-dimensional (2D) materials.

2D Material	Nanostructured Films	Epitaxial Films	Applications
MoS_2_	Defect-enhanced magnetism	Uniform, low defects	Spintronics, sensors
WS_2_	Quantum confinement effects	Smooth, ordered	Optoelectronics
h-BN	Tunable vacancy sites	High-quality dielectric	Substrates, tunneling barriers

**Table 5:** Recent Advances in Nanostructured Perovskite Thin Films.

Type of Perovskite/System	Article	Novelty Statement
CsPbBr_3_ perovskite thin films	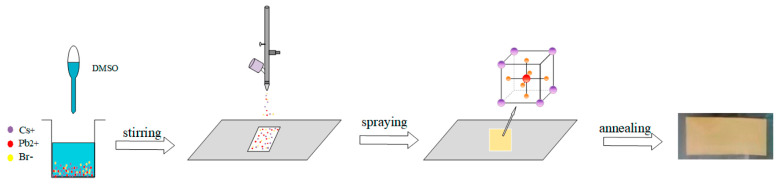	Introduced an ambient, one-step ion-spray method enabling scalable fabrication of high-quality CsPbBr_3_ perovskite thin films with tunable microstructure [152].
Lead-free CsBi_3_I_0_ perovskite thin films	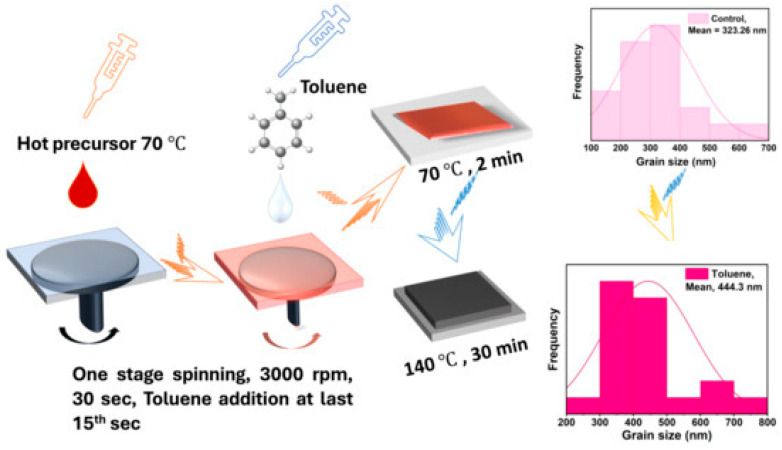	A novel approach to creating lead-free perovskite thin films, focusing on enhancing the optoelectronic properties of cesium bismuth iodide (CsBi_3_I_10_) perovskites by employing antisolvent-assisted crystallization techniques [153].
Multilayered CH_3_NH_3_PbIBr_2_ perovskite thin films	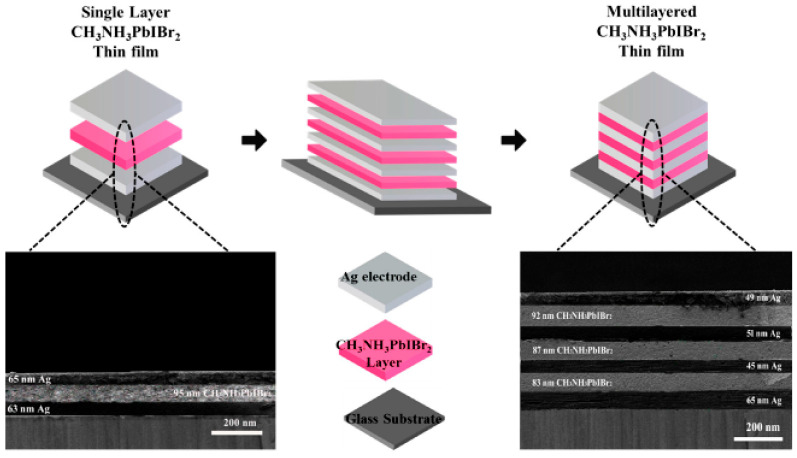	Multilayered CH_3_NH_3_PbIBr_2_ perovskite thin films achieve enhanced crystallinity, larger grain sizes, reduced defects, and higher photovoltaic efficiency (~13.8%) compared to single-layer films, demonstrating that multilayer architectures directly improve optoelectronic performance and stability [154].
d-MAPbI3-HEA and d-FAPbI3-TEA perovskite thin films	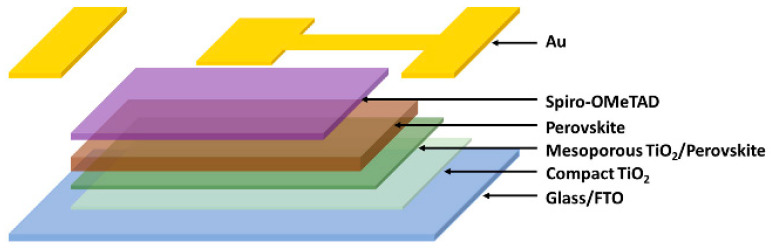	Introducing lead- and iodide-deficient (d-HP) perovskite thin films that exhibit enhanced stability and tunable optoelectronic properties, enabling a better balance between efficiency and environmental resilience compared to standard stoichiometric perovskite films [155].

**Table 6:** Tailoring Nanostructured Thin-Film Properties: Methods and Outcomes.

Property to Be Tailored	Approach/Method	Observed Effect	Also Affected	References
Refractive index	Modify nanoporosity	Higher porosity → smaller refractive index	Air-to-vacuum spectral shifts, mechanical properties, light scattering	[54,55]
Catalytic activity	Control grain size, surface morphology	Increased active site density → improved catalytic performance	Adsorption/desorption kinetics, phase stability	[40,41,42,43]
Bandgap/optical absorption	Reduce nanostructure size (quantum confinement)	Bandgap widening, altered absorption/emission spectra	Charge carrier mobility, exciton dynamics	[52,53]
Electrical conductivity	Annealing/grain growth	Enhanced carrier mobility, lower resistivity	Transparency, defect-state distribution	[62,63,64,65,66,67,68,69,70,71,72]
AFM → FM transition temperature	Doping (Co, Pd, Ir)	Tunable magnetic phase transition temperature	Electrical resistivity, magnetic anisotropy	[86,87,88,89,90]
Surface-enhanced Raman scattering (SERS)	Nanostructure patterning/porous metal deposition	Strong electromagnetic hot-spots → enhanced Raman signal	Morphology-dependent reproducibility, plasmon resonance shifts	[66,67]
Photocatalytic activity	Introduce surface defects/porosity	Enhanced charge separation → higher photocatalytic efficiency	Bandgap modulation, surface stability	[47,48,49]
Magnetic anisotropy (PMA)	Control thickness/interface engineering	Transition from in-plane to perpendicular anisotropy	Spin–orbit coupling, domain structure, coercivity	[83,84]
Surface energy/reactivity	Reduce particle size/increase surface-to-volume ratio	Higher surface energy → improved reactivity	Phase transitions at lower temperature, densification	[34,35,36,37,38,39]
Gas sensing	Introduce surface defects or dopants	Increased adsorption → higher sensitivity	Selectivity, response/recovery kinetics	[40,100]
Ion diffusion/conductivity	Increase grain boundary networks/nanoscale porosity	Enhanced ionic transport	Mechanical stability, film density	[45,46]

## Data Availability

Data are contained within the article.
